# Viability and Virulence of *Listeria monocytogenes* in Poultry

**DOI:** 10.3390/microorganisms11092232

**Published:** 2023-09-04

**Authors:** Sarah Panera-Martínez, Rosa Capita, Camino García-Fernández, Carlos Alonso-Calleja

**Affiliations:** 1 Department of Food Hygiene and Technology, Veterinary Faculty, University of León, 24071 León, Spain; 2 Institute of Food Science and Technology, University of León, 24071 León, Spain

**Keywords:** *Listeria monocytogenes*, quantification, viable non-culturable cells, virulence, biofilm, antibiotic resistance

## Abstract

The prevalence of *Listeria monocytogenes* in 30 samples of poultry was determined using culture-dependent (isolation on OCLA and confirmation by conventional polymerase chain reaction -PCR-, OCLA&PCR) and culture-independent (real-time polymerase chain reaction, q-PCR) methods. *L. monocytogenes* was detected in 15 samples (50.0%) by OCLA&PCR and in 20 (66.7%) by q-PCR. The concentrations (log_10_ cfu/g) of *L. monocytogenes* (q-PCR) ranged from 2.40 to 5.22 (total cells) and from <2.15 to 3.93 (viable cells). The two methods, q-PCR using a viability marker (v-PCR) and OCLA&PCR (gold standard), were compared for their capacity to detect viable cells of *L. monocytogenes*, with the potential to cause human disease. The values for sensitivity, specificity and efficiency of the v-PCR were 100%, 66.7% and 83.3%, respectively. The agreement between the two methods (kappa coefficient) was 0.67. The presence of nine virulence genes (*hlyA*, *actA*, *inlB*, *inlA*, *inlC*, *inlJ*, *prfA*, *plcA* and *iap*) was studied in 45 *L. monocytogenes* isolates (three from each positive sample) using PCR. All the strains harbored between six and nine virulence genes. Fifteen isolates (33.3% of the total) did not show the potential to form biofilm on a polystyrene surface, as determined by a crystal violet assay. The remaining strains were classified as weak (23 isolates, 51.1% of the total), moderate (one isolate, 2.2%) or strong (six isolates, 13.3%) biofilm producers. The strains were tested for susceptibility to a panel of 15 antibiotics. An average of 5.11 ± 1.30 resistances per isolate was observed. When the values for resistance and for reduced susceptibility were taken jointly, this figure rose to 6.91 ± 1.59. There was a prevalence of resistance or reduced susceptibility of more than 50.0% for oxacillin, cefoxitin, cefotaxime, cefepime ciprofloxacin, enrofloxacin and nitrofurantoin. For the remaining antibiotics tested, the corresponding values ranged from 0.0% for chloramphenicol to 48.9% for rifampicin. The high prevalence and level of *L. monocytogenes* with numerous virulence factors in poultry underline how crucial it is to follow correct hygiene procedures during the processing of this foodstuff in order to reduce the risk of human listeriosis.

## 1. Introduction

The genus *Listeria* is composed of Gram-positive, rod-shaped, facultatively anaerobic, psychrotrophic bacteria that do not form spores [1]. Although, in total, 26 species of *Listeria* have been described [2], the most prevalent species in food are *Listeria monocytogenes*, *Listeria innocua*, *Listeria grayi*, *Listeria seeligeri* and *Listeria ivanovii* [3,4]. *L. monocytogenes* is the species responsible for the vast majority of cases of listeriosis in both humans and animals [5,6]. Listeriosis is a zoonosis associated with a high fatality rate and is mostly contracted by consuming contaminated food [7,8]. This bacterium can be found in different types of foodstuffs, including meat, milk, vegetables and even ready-to-eat (RTE) foods [9].

Culture-dependent methods based on two stages of enrichment in a liquid medium, with the subsequent isolation and identification of the colonies by biochemical or molecular methods, have been commonly used for the detection of *L. monocytogenes* in samples of food origin [10]. Such methods are incorporated into European Union regulations, specifically Commission Regulation (EC) No. 2073/2005. Detection can also be performed using rapid methods based on molecular techniques such as q-PCR, requiring less time to obtain results, having greater specificity, and also permitting the detection of viable but non-culturable cells [11,12]. In view of their potential pathogenicity for consumers, *L. monocytogenes* cells in a viable, non-culturable state pose a challenge for food safety and public health.

However, q-PCR is not a widely used technique for quantifying bacteria, since it may overestimate bacterial concentrations because of the presence and detection of DNA from dead cells [13,14]. In recent years, a variant of q-PCR, viability PCR (v-PCR), has become increasingly common in various sectors, such as food safety, with this method allowing for cell viability to be determined [15]. The technique is based on treating samples with a viability marker, for example, propidium mono-azide (PMA) [16]. This compound is only capable of penetrating inactivated bacteria with damaged cell membranes. Once inside, the marker binds to the DNA molecules, preventing their amplification during the subsequent PCR process [17], consequently achieving only the selective amplification of the DNA of the viable cells [18,19]. In this way, if q-PCR is performed with and without PMA, viable cells and total cells can be detected and quantified separately.

The virulence of a pathogenic microorganism is related to its ability to cause death in an infected host [20,21,22]. Different virulence factors can be considered, such as the inherent components of the microorganisms that cause damage to host cells, for example, through the production of endotoxins, or the mechanisms that allow a microorganism to evade host defense systems, such as a capacity to form biofilm or resistance to antibiotics [23].

Checks on meat from poultry are a fundamental aspect of food safety, in view of the worldwide trend toward increased consumption of this type of food [12,24]. In this context, this research work was undertaken with the objective of determining the prevalence of *L. monocytogenes* in poultry meat cuts, quantifying the cells present in both viable and inactivated physiological states. In addition, three factors related to the virulence of the strains, namely, the presence of virulence genes, the potential of the bacteria to form biofilms, and their resistance to antibiotics, were also investigated.

## 2. Materials and Methods

### 2.1. Samples

Thirty samples (of approximately 300 g each) of poultry meat cuts (chicken and turkey) were obtained from nine retail outlets (E1 to E9) in the city of León in the North-West of Spain. From one to seven samples were purchased, depending on the establishment concerned: E1 (4 samples), E2 (1), E3 (7), E4 (1), E5 (1), E6 (6), E7 (4), E8 (2), E9 (4). The cuts analyzed included wings (11 samples), thighs (3), drumsticks (12) and breasts (4). All samples were transported in individual bags and processed immediately upon arrival at the laboratory.

### 2.2. Isolation and Identification of Listeria spp.

Twenty-five grams of skin were homogenized with 225 mL of Half-Fraser broth in sterile bags for two minutes, using a Masticator (IUL Instruments, Barcelona, Spain). These bags were then incubated at 30 °C. After 24 h, aliquots comprising 100 µL were taken and transferred to tubes with 10 mL of Fraser broth, then incubated at 37 °C for a further 24 h. Subsequently, cultures were streaked onto plates of Oxoid chromogenic *Listeria* agar (OCLA), then incubated at 37 °C for 48 h, in accordance with the ISO 11290-1 standard. From each positive sample, three colonies with attributes typical of *Listeria* spp. (green colonies) and/or three colonies with those specifically characteristic of *L. monocytogenes* (green colonies with halo) were taken. The strains isolated were stored at −50 °C in tryptone soy broth (TSB) with 20% glycerol. All the culture media used were purchased from Oxoid Ltd. (Hampshire, UK).

Identification of the isolates was achieved by conventional PCR. This involved growing the strains in TSB at 37 °C for 24 h, and thereafter extracting the DNA from 1.5 mL of the culture. This extraction was performed using two cycles of centrifugation at 13,000 rpm for 60 s, then exposure in a water bath at 100 °C for 30 min. The purity and concentration of the DNA were determined with a NanoDrop™ One spectrophotometer (ThermoFisher Scientific, Wilmington, DE, USA), a wavelength of 260 nm being used. Samples with a DNA concentration between 80 and 180 ng/µL were deemed valid for analysis.

The target genes used for identification were specific for *L. monocytogenes* (*lmo1030*), *L. innocua* (*lin0464*), *L. grayi* (*oxidoreductase*), *L. seeligeri* (*lmo0333*), *L. ivanovii* (*namA*) and *Listeria* spp. (*prs*), as shown in Table 1 [9]. The amplification reaction was performed in a total volume of 25 µL, including 5 µL of the extracted DNA, 2.50 µL of incomplete NH_4_ reaction buffer (10×, BIORON GmbH, Ludwigshafen, Germany), 1.50 µL of MgCl_2_ (25 mM, BIORON), 0.50 µL of dNTP mix (10 mM, EURx, Gdansk, Poland), 0.50 µL of each primer (25 µM, Macrogen, Seoul, Republic of Korea), 0.25 µL of Taq DNA polymerase (5 U/µL, BIORON) and 14.25 µL of molecular biology grade distilled water.

Amplification reactions were carried out in a ProFlex™ thermal cycler (Applied Biosystems, Waltham, MA, USA). Denaturation was carried out for five minutes at 94 °C and subsequently, 35 amplification cycles were performed. Each comprised: denaturation for 30 s at 94 °C, annealing for 30 s at the temperature required for each primer, as indicated in Table 1, and elongation for 45 s at 72 °C. As a last stage, there was an extension period of five minutes at 72 °C. Positive controls (previously identified strains), and negative controls (samples without any DNA) were included.

The products of PCR were separated by horizontal electrophoresis in 1% agarose gel (BIORON) dissolved in Tris-acetate-EDTA buffer at 1× concentration and stained with SimplySafe (EURx,) diluted at 1× concentration. The results were visualized using a Gel Doc™ EZ System ultraviolet transilluminator (Bio-Rad, Hercules, CA, USA) and the size of each amplified fragment was estimated using a standard molecular weight marker (Perfect Plus 1kb DNA Ladder, EURx).

### 2.3. Quantification and Viability of Listeria monocytogenes Determined by q-PCR

Twenty-five grams of skin was homogenized with a Masticator (IUL Instruments) for two minutes in 225 mL of 0.1% peptone water. Two one-milliliter aliquots were separated from each homogenate, one to determine the total cell concentration and the other exclusively for viable cells (v-PCR). This second aliquot was treated prior to DNA extraction with 25 µM of PMAxx™ dye (Biotium, Landing Parkway, Fremont, CA, USA), which is only capable of penetrating inactivated bacteria with damaged cell membranes, where it binds DNA molecules and prevents their amplification.

After this procedure, DNA extraction was performed on the two aliquots, with and without PMAxx, using a commercial protocol PrepSEQ™ Rapid Spin Sample Preparation Kit with proteinase K (ThermoFisher Scientific, Waltham, MA, USA). For this purpose, 750 µL of each aliquot was loaded into separate extraction columns, and centrifuged at 13,000 rpm for three minutes. The column and supernatant were discarded, while the pellet was resuspended in 50 µL of buffer during lysis with proteinase K, incubated in a heat block for 30 min at 56 °C and then 12 min at 97 °C, in order to inactivate the proteinase K. After further centrifuging at 13,000 rpm for one minute and the addition of 250 µL of Milli-Q water to make up a total volume of 300 µL, each sample was centrifuged once more at 13,000 rpm for two minutes, leaving the DNA suspended in the aqueous phase.

Amplification by q-PCR was performed in a StepOne™ thermal cycler (Applied Biosystems, Foster City, CA, USA) using the commercial package MicroSEQ™ *L. monocytogenes* Detection Kit (ThermoFisher Scientific). The amplification results were transformed into the amount of DNA of the microorganism by setting a fluorescence threshold of 0.3, and using a standard straight line (y = −3.0525 x + 23.206; R^2^ = 0.966) previously calculated from samples with known amounts of DNA from *L. monocytogenes*. In order to transform the amount of DNA into log_10_ cfu/g of the sample, the following equation was used [12]:

$$Concentration={log}_{10}\left(\frac{10^{\frac{Ct-23.206}{-3.0525}}\times 340,000\times 10^{5}}{750}\right)cfu/g$$

In arriving at this formula, several factors were taken into account: (1) the total volume of the homogenization bag (250 mL); (2) the decimal dilution (10^−1^) of the homogenization bag (25 g of sample in 225 mL of diluent); (3) the fact that one-tenth of the total amount of DNA extracted, 30 µL out of a total of 300 µL, was placed in the reaction tube; (4) the fact that the DNA was extracted from a volume of 750 µL; and (5) the size of the *L. monocytogenes* genome, as 1 ng of DNA equates to approximately 340,000 cfu [25].

Finally, the percentage of viable cells in each sample was determined by comparing the concentration values obtained from the aliquot of viable cells, treated with PMA, and in the aliquot of total cells, not so treated, using the following formula:

$$\%viable\;cells=\frac{Concentration\;in\;aliquot\;with\;PMA\left(\frac{cfu}{g}\right)}{Concentration\;in\;aliquot\;without\;PMA\;\left(\frac{cfu}{g}\right)}\times 100$$

### 2.4. Comparison between OCLA&PCR and v-PCR Techniques

**Figure 1:**
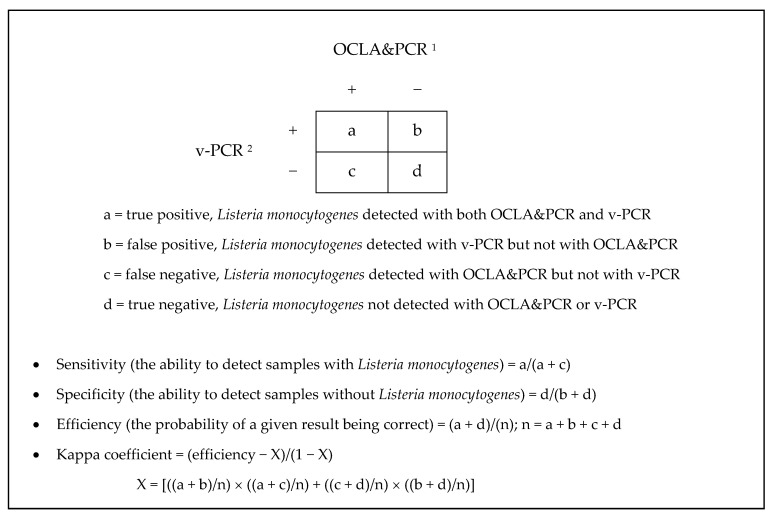
Definitions and calculations for sensitivity, specificity, efficiency, and kappa coefficient for the v-PCR technique used in detecting viable cells of *Listeria monocytogenes* in poultry: (1) OCLA&PCR, isolation using the culture-dependent method ISO 11290-1 involving streak plating on OCLA and identification of presumptive colonies by polymerase chain reaction (PCR); (2) quantitative PCR detection (v-PCR), using the viability marker propidium monoazide (PMAxx).

The two methods used for detecting viable *L. monocytogenes* cells were compared, the first being the classic method involving isolation in OCLA medium (ISO 11290-1) followed by identification using PCR (OCLA&PCR), the second the culture-independent method (v-PCR). Since the prevalence of positive samples was unknown, it was assumed that the conventional method (OCLA&PCR) would provide the correct results, since it is considered to be the reference technique, or “gold standard”. The sensitivity, specificity, efficiency, and predictive values in respect of both positive and negative tests of the culture-independent method (v-PCR) were calculated. In addition, the two methods were compared by calculating the agreement between them in terms of their kappa coefficient [26]. The definitions and calculations for these parameters are shown in Figure 1.

### 2.5. Virulence Genes

From the same DNA extractions used to carry out the identification of the isolates, the presence or absence of nine major virulence factors was determined for *L. monocytogenes*, as indicated in Table 2 [27].

The *hlyA*, *actA*, *inlB* and *iap* genes were detected by single PCRs, while two multiple PCRs were performed for the remaining virulence genes: one for *inlA*, *inlC* and *inlJ*, and another for *plcA* and *prfA*. Single PCRs were performed using the same reagent concentrations employed to identify isolates (Table 1). However, the primers used, their concentrations and the thermocycling conditions were those shown in Table 3. In the case of the multiplex PCRs, a final volume of 50 µL was obtained, and the concentrations of MgCl_2_ (2 mM), of Taq DNA polymerase (2U), and of each primer used were modified, as can be seen in Table 2.

Amplification reactions were carried out in a ProFlex™ thermal cycler (Applied Biosystems). Results were examined by electrophoresis in 1.5% agarose gel and visualized by means of a UV light transilluminator (Bio-Rad).

### 2.6. Ability to Form Biofilm

Determination of the capacity of *L. monocytogenes* to form biofilm was performed in accordance with a protocol previously described by Díez-García et al. [28]. For this purpose, strains preserved at −50 °C in TSB with 20% glycerol were inoculated into tubes of TSB and incubated for 18 h at 37 °C, yielding a concentration of approximately 10^9^ cfu/mL. Four decimal dilutions were carried out to obtain a concentration of 10^5^ cfu/mL. Volumes of 225 µL of TSB and 25 µL of bacterial culture were deposited in the wells of polystyrene microtiter plates (Oy Growth Curves Ab Ltd., Helsinki, Finland), so as to reach a final concentration in the wells of 10^4^ cfu/mL. Negative controls, containing 250 µL of plain TSB, were included on all the plates.

The microtiter plates were incubated at 37 °C for 24 h, after which the culture broth was drawn off and the wells were washed with 300 µL of sterile distilled water. The bacteria that remained adhering to the bottom of each well were fixed with 250 µL of methanol for 15 min. After this time had elapsed, the methanol was poured off, the plates were air dried, and then stained through five minutes of contact with 250 µL of a 0.5% aqueous crystal violet solution. The wells were then emptied and washed with running tap water. The plates were air-dried once again and the cell-bound dye was resolubilized by adding 250 µL of 33% acetic acid (Sigma-Aldrich Co., St. Louis, MO, USA) to the wells for subsequent measurement of the optical density at 580 nm (OD_580_) with a Bioscreen C MBR (Oy Growth Curves Ab).

Finally, the strains were classified on the basis of their ability to form biofilms, using a cut-off optical density at 580 nm, OD_580_, designated ODc. This ODc was defined as the mean OD_580_ value for negative controls plus three standard deviations. In this way, the strains were divided into four categories: non-biofilm producers, for which OD_580_ ≤ ODc; weak biofilm producers (ODc < OD_580_ ≤ 2 × ODc); moderate biofilm-producers (2 × ODc < OD_580_ ≤ 4 × ODc); and strong biofilm producers (4 × ODc < OD_580_) [29].

### 2.7. Antibiotic Resistance

The susceptibility of all the *L. monocytogenes* strains taken from the OCLA medium and identified by PCR was tested against 15 antibiotics of clinical importance using the disc diffusion technique [30]. The strains were incubated at 37 °C in Mueller–Hinton broth (MHB). Thereafter, they were inoculated onto dishes of Mueller–Hinton agar (MHA) by spread plating, after which antibiotic discs were placed onto the dishes, with five antibiotics on each.

The antibiotic discs (Oxoid) used were ampicillin (AMP, 10 µg), oxacillin (OX, 1 µg), cefoxitin (FOX, 30 µg), cefotaxime (CTX, 30 µg), cefepime (FEP, 30 µg), gentamycin (CN, 10 µg), erythromycin (E, 15 µg), vancomycin (VA, 30 µg), trimethoprim-sulfamethoxazole (SXT, 25 µg), rifampicin (RD, 5 µg), tetracycline (TE, 30 µg), chloramphenicol (C, 30 µg), ciprofloxacin (CIP, 5 µg), enrofloxacin (ENR, 5 µg) and nitrofurantoin (F, 300 µg). After incubation at 37 °C for 18–24 h, inhibition halos were measured and the strains were classified as being susceptible, as intermediate (with reduced susceptibility), or as resistant on the basis of the same criteria used in previous work [12]. These norms were (1) those of EUCAST [31] for E, SXT (*L. monocytogenes*), CN, RD, TE, C, CIP (*Staphylococcus* spp.), CTX, FEP (*Streptococcus* spp.) and VA (*Enterococcus* spp.); (2) those of the CLSI [30] in the case of OX, FOX, F (*Staphylococcus* spp.), AMP (*Enterococcus* spp.); and (3) the CLSI’s VET08 standard [32] for ENR (*Staphylococcus* spp.).

Finally, with these data, antibiotic resistance patterns were established on the basis of the criteria defined by the European Centre for Disease Prevention and Control (ECDC) and the Centers for Disease Control and Prevention (CDC) of the USA for classifying bacteria of interest for public health. These norms include references to “multidrug-resistant” (MDR), “extensively drug-resistant” (XDR) and “pan-drug-resistant” (PDR) phenotypes. The MDR phenotype is defined as an acquired absence of susceptibility to at least one antibiotic from each of three or more categories of antimicrobials. The XDR phenotype is explained as a lack of susceptibility to at least one antimicrobial agent from all but two or fewer antimicrobial categories. Finally, the PDR phenotype involves an absence of susceptibility to all agents in all antimicrobial categories [33].

### 2.8. Statistical Analysis

The data obtained, both for the prevalence of bacteria as well as for the prevalence of virulence genes and the percentages of resistance to antibiotics, were compared using exact Chi-square tests. To determine if there were differences between the quantification results for samples that had been treated with PMA and those that had not, a Mann–Whitney U test was performed. In addition, a Pearson correlation analysis was performed between the variables studied: the percentage of virulence genes relative to the total number of virulent genes tested shown by each isolate, the capacity to form biofilm, OD_580_ values, and the percentage of reactions of resistance to antibiotics relative to the total number of tests carried out for each isolate. Outcomes were grouped into four categories: no correlation (0.00 ≤ r < 0.10), weak correlation (0.10 ≤ r < 0.30), moderate correlation (0.30 ≤ r < 0.50) and strong correlation (0.50 ≤ r < 1.00), on lines similar to what is described by Cohen [34] and Hernández-Lalinde et al. [35]. All analyses were carried out using the RStudio software package, V.3.6.3 [36], and the Statistica^®^ 8.0 package (Statsoft Ltd., Tulsa, OK, USA), the confidence level being set at 95.0% (*p* < 0.05).

## 3. Results

### 3.1. Prevalence of Listeria spp. and Listeria monocytogenes

When the OCLA&PCR method (isolation on OCLA and confirmation by conventional PCR), dependent upon culturing, was used, *Listeria* spp. cells were detected in 21 samples (70.0%). In nine of these, *L. monocytogenes* was the species detected, in four, *L. innocua*, and in seven, more than one species was found. The combinations were *L. monocytogenes* and *L. innocua* (five samples), *L. innocua* and *L. grayi* (one), and *L. monocytogenes*, *L. innocua* and *L. grayi* (one). Isolates from one of the samples were assignable to the genus *Listeria*, but could not be identified at the species level. Hence, *L. monocytogenes* was detected in 50.0% of the samples analyzed, and from each of these positive samples, three colonies were isolated, yielding 45 isolates in total.

**Figure 2:**
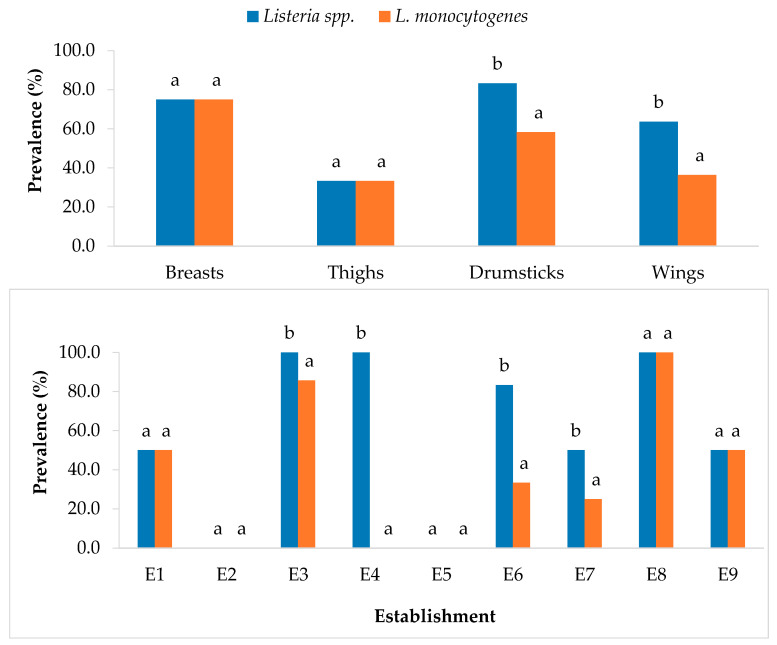
Prevalence of *Listeria* spp. and *Listeria monocytogenes* relative to type of sample (upper graph) and establishment where purchased (lower graph). The columns in the same graph for the same type of sample or the same establishment (the prevalence values for *Listeria* spp. and *L. monocytogenes* were compared) that do not share any letter present significant differences one from another (*p* < 0.05).

Figure 2 shows the prevalence of *Listeria* spp. and *L. monocytogenes* in respect of the particular type of poultry cut and the outlet where it was purchased. In the case of *Listeria* spp., thighs were the samples with a tendency to show the lowest prevalence of *Listeria* spp. (33.3%), while for other cuts, the prevalence ranged between 63.6% for wings and 83.3% for breasts. In establishments E2 and E5, no sample with *Listeria* spp. was detected, while in the remaining outlets, between 50% and 100% of the samples were contaminated with this microorganism.

With regard to the prevalence specifically of *L. monocytogenes*, the samples with a tendency to show the highest prevalence of *L. monocytogenes* were drumsticks (58.3%) and breasts (75.0%). Furthermore, establishments E2, E4 and E5 had no samples among those they provided that were contaminated with *L. monocytogenes*. In contrast, the outlets labeled E3 and E8 showed the highest figures for contamination with this microorganism, the first having 85.7%, and the second, 100% of all the items purchased.

Similar (*p* > 0.05) prevalence values were observed for *Listeria* spp. and *L. monocytogenes* in breasts and thighs. On the other hand, drumsticks and wings showed the highest (*p* < 0.05) prevalence for *Listeria* spp. With regard to the establishment, differences in the prevalence of *Listeria* spp. and *L. monocytogenes* were observed for E3, E4, E6 and E7.

### 3.2. Concentration of Viable Cells of Listeria monocytogenes

Table 4 shows the levels of *L. monocytogenes* obtained using q-PCR. It indicates the concentrations of total cells and viable cells, as well as the percentage of viable cells in each of the samples, and the results, whether positive or negative, obtained with the culture-dependent OCLA&PCR method. In cases where no amplification was observed in the samples treated with PMA, the percentage of viable cells was calculated on the basis of the figure for 40 amplification cycles (detection limit).

**Figure 3:**
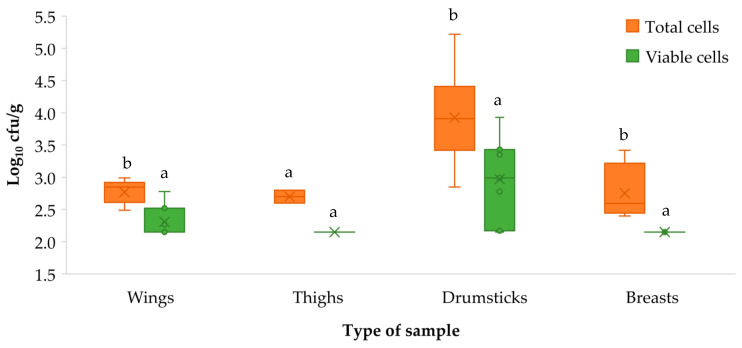
Box and Whisker graph showing concentrations of *Listeria monocytogenes* (log_10_ cfu/g) by type of sample and treatment used: total cells (without PMA) and viable cells (with PMA). The boxes run from the 25 to the 75 percentile and are intersected by the median line. The average value is marked inside each box with a cross (×). The whiskers range from the lowest to the highest value of each sample. The values for the same type of sample (concentrations of total cells and viable cells were compared) not sharing any letter present significant differences (*p* < 0.05).

There were significant differences (*p* < 0.001) between the concentrations of *L. monocytogenes* (log_10_ cfu/g) in the aliquots not treated with PMA, for which the average concentration of total cells was 3.16 ± 0.74, and those that were treated, where the mean concentration of viable cells was 2.47 ± 0.54. Analysis of these data in accordance with the type of sample involved yielded the results shown in Figure 3. Significant differences *p* < 0.05) were observed between total cells (before treatment with PMA) and viable cells (after this treatment) in the case of wings, drumsticks and breasts.

**Figure 4:**
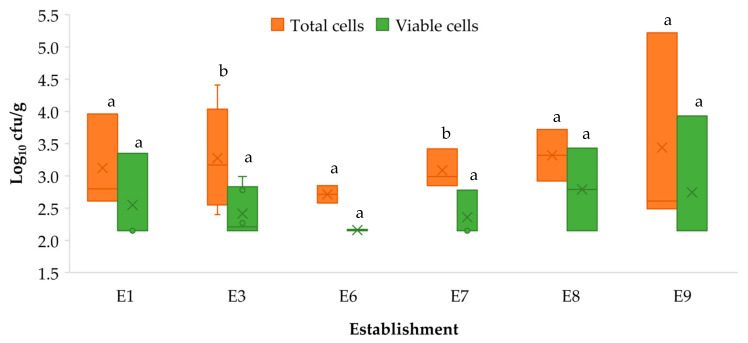
Box and Whisker graph showing concentrations of *Listeria monocytogenes* (log_10_ cfu/g) by establishment and treatment used: total cells (without PMA) and viable cells (with PMA). The boxes run from the 25 to the 75 percentile and are intersected by the median line. The average value is marked inside each box with a cross (×). The whiskers range from the lowest to the highest value for each sample. Establishments E2, E4 and E5 were excluded because no positive samples (E2 and E4) or only one positive sample (E5) were found by q-PCR. The values for the same type of sample (concentrations of total cells and viable cells were compared) not sharing any letter present significant differences (*p* < 0.05).

Regarding the establishment (Figure 4), significant differences (*p* < 0.05) between total and viable cells were found in E3 and E7. As previously noted, establishments E2, E4 and E5 provided no samples positive for *L. monocytogenes* and, thus, were excluded from the statistical analysis.

### 3.3. Comparison between OCLA&PCR and v-PCR

When the capacity of the two methods (OCLA&PCR and v-PCR) used for the detection of *L. monocytogenes* in poultry cuts was compared, taking OCLA&PCR as the reference method, the v-PCR technique obtained sensitivity values of 100%, specificity of 66.7% and efficiency of 83.3%. The agreement between the two methods (kappa coefficient) was 0.67. All the samples found positive for *L. monocytogenes* by the OCLA&PCR method, 15 in total, were also recorded as positive by the v-PCR technique. However, five samples showed positive for the pathogen with v-PCR, but not so with the classic method (OCLA&PCR). Finally, ten samples were rated negative by both methods.

### 3.4. Virulence Genes

**Figure 5:**
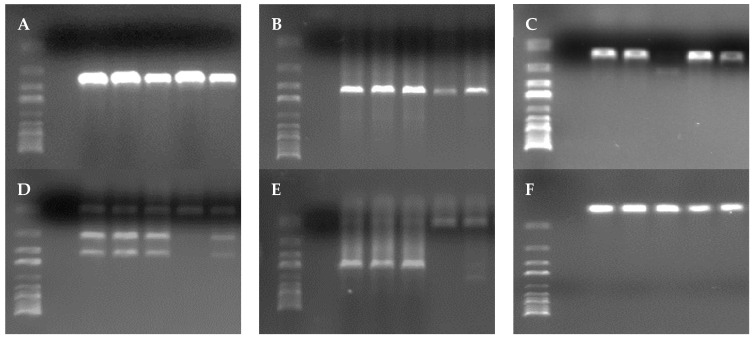
Agarose gels (1.5%) with results from PCR. The genes are (**A**) *hlyA*, (**B**) *actA*, (**C**) *inlB*, (**D**) *inlA, inlC* and *inlJ*, (**E**) *prfA* and *plcA*, and (**F**) *iap.* Within each image (**A**–**F**) are displayed 7 lanes, from left to right: the size marker (from 0.25 to 10 kb), the negative control and the results obtained from five isolates.

The presence of nine virulence genes, *hlyA*, *actA*, *inlB*, *inlA*, *inlC*, *inlJ*, *prfA*, *plcA* and *iap*, was investigated using PCR in the 45 isolates identified as *L. monocytogenes*, as shown in Figure 5. All the strains studied were positive for between six and nine virulence genes. A total of 22.2% of the isolates were positive for all nine genes studied, 62.2% for eight, 13.3% for seven, and 2.2% for six genes.

**Figure 6:**
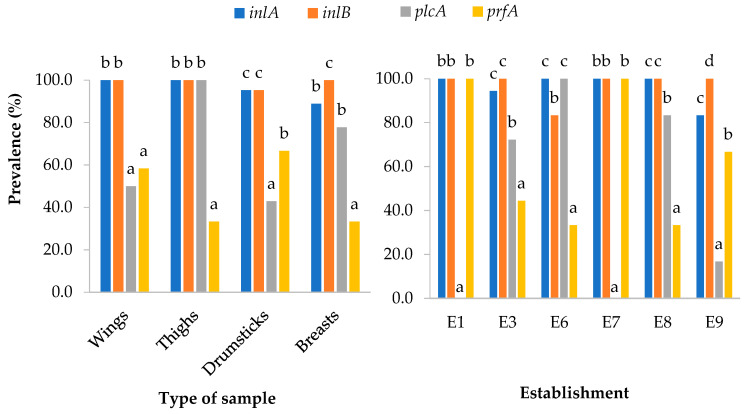
Percentage of samples having virulence genes *inlA*, *inlB*, *plcA* and *prfA* by type of sample (left-hand graph) and outlet (right-hand graph). Columns in each graph for the same type of cut or the same establishment (the prevalence of the four virulence genes were compared) not sharing any letter present significant differences one from another (*p* < 0.05). No *L. monocytogenes* strains were isolated from establishments E2, E4 and E5.

All the strains analyzed, regardless of the type of sample or the establishment where it was obtained, were positive for the *hlyA*, *actA*, *inlC*, *inlJ* and *iap* genes. The lowest percentage of prevalence was observed in the case of the *plcA* and *prfA* genes, present in 55.6% of the isolates in both cases. The *inlA* gene was found in 95.6% of the isolates and *inlB* in 97.8%. Figure 6 shows the prevalence of each gene according to the type of cut and the establishment. In most cases, the highest prevalence (*p* < 0.05) was observed for *inlA* and *inlB* genes.

### 3.5. Potential for Biofilm Formation

Fifteen isolates (33.3% of the total) did not form biofilm on the polystyrene surfaces used. The remaining strains had a potential for biofilm formation, and were classified as weak (23 isolates, 51.1% of the total), moderate (one isolate, 2.2%) or strong (six isolates, 13.3%) biofilm producers. The average OD_580_ of the negative controls was 0.134 ± 0.073. Consequently, in classifying strains the ODc was set at 0.353.

**Figure 7:**
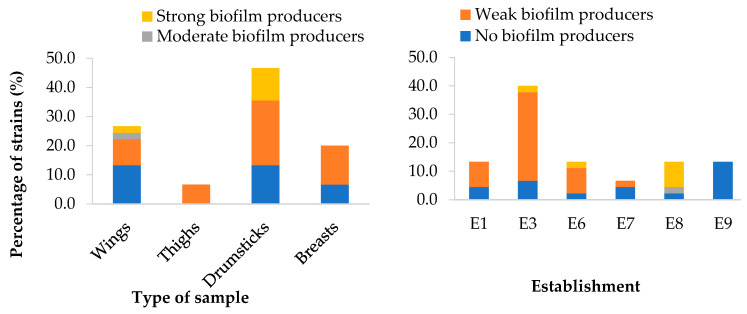
Percentages of *Listeria monocytogenes* isolates classified on the basis of their potential for biofilm formation, by type of sample (left-hand graph) and by establishment (right-hand graph).

Figure 7 shows the distribution of strains on the basis of their potential to form biofilm, in respect of the type of sample and the establishment of acquisition. All the strains classified as strong biofilm producers were isolated from wings or drumsticks from the outlets labeled E3, E6 and E8, while those classified as moderate biofilm producers were isolated from wings purchased in establishment E8.

The overall average value for OD_580_ obtained from the whole set of isolates was 0.639 ± 0.594. According to the type of cut, OD_580_ values were 0.542 ± 0.433 (wings), 0.306 ± 0.141 (thighs), 0.493 ± 0.433 (drumsticks) and 1.220 ± 0.849 (breasts). Data for establishments were 0.434 ± 0.121 (E1), 0.507 ± 0.366 (E3), 0.662 ± 0.398 (E6), 0.333 ± 0.159 (E7), 1.730 ± 0.844 (E8) and 0.278 ± 0.053 (E9).

### 3.6. Antibiotic Resistance

The 45 isolates of *L. monocytogenes* were tested for susceptibility to a panel of 15 antibiotics. An average of 5.11 ± 1.30 resistances per isolate was recorded. When figures for resistance and reduced susceptibility were taken together, this number rose to 6.91 ± 1.59.

**Figure 8:**
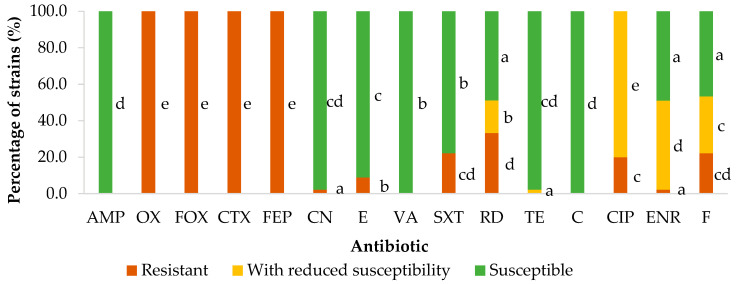
Percentage of isolates of *Listeria monocytogenes* showing resistance, reduced susceptibility or susceptibility to each antibiotic tested. AMP (ampicillin, 10 µg), OX (oxacillin, 1 µg), FOX (cefoxitin, 30 µg), CTX (cefotaxime, 30 µg), FEP (cefepime, 30 µg), CN (gentamycin, 10 µg), E (erythromycin, 15 µg), VA (vancomycin, 30 µg), SXT (trimethoprim-sulfamethoxazole, 25 µg), RD (rifampicin, 5 µg), TE (tetracycline, 30 µg), C (chloramphenicol, 30 µg), CIP (ciprofloxacin, 5 µg), ENR (enrofloxacin, 5 µg) and F (nitrofurantoin, 300 µg). Columns of the same color (resistant strains, those with reduced or intermediate susceptibility, and susceptible strains are compared separately) not sharing any letter present significant differences one from another (*p* < 0.05).

Consideration of average resistance data for the antibiotics examined, as shown in Figure 8, revealed significant differences (*p* < 0.05) between substances. All the strains analyzed were resistant to OX, FOX, CTX and FEP, but none were resistant to AMP, VA, TE and C. For the rest of the antibiotics, the percentages of resistance ranged from 2.2% (CN and ENR) to 33.3% (RD). If resistant strains are lumped together with those having reduced susceptibility, prevalence was higher than 50.0% for OX, FOX, CTX, FEP, CIP, ENR and F. For all the other antibiotics, with the exception of C (0.00%), there was some prevalence, with values ranging from 2.2% (TE) to 48.9% (RD).

Seventeen different resistance patterns were found, as displayed in Table 5. Two main phenotypes were prominent: OX-FOX-CTX-FEP (presented by 19 isolates) and OX-FOX-CTX-FEP-RD (six isolates). The other patterns were present in a number of isolates ranging from one to three.

**Figure 9:**
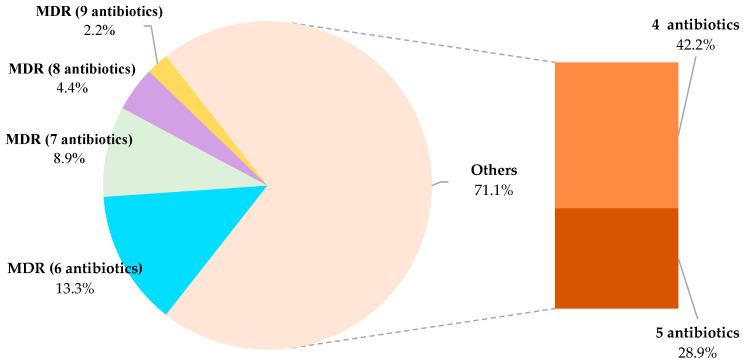
Isolates of *Listeria monocytogenes* grouped by number of antibiotic resistances found. To the left are the strains with an MDR phenotype, separated according to the number of antibiotics to which they were resistant, and to the right are the strains having resistance to only two different categories of antibiotics. MDR is defined as an acquired non-susceptibility to at least one antibiotic in three or more categories of antimicrobials, one or more of which must be applied.

A total of ten different categories of antibiotics were tested: beta-lactams (AMP, OX, FOX, CTX and FEP), aminoglycosides (CN), macrolides (E), glycopeptides (VA), sulphonamides (SXT), rifamycins (RD), tetracyclines (TE), phenicols (C), fluoroquinolones (CIP, ENR) and nitrofurans (F). Of the forty-five strains tested, none had a phenotype that was pan-drug-resistant (PDR) or extensively drug-resistant (XDR, implying resistance to between eight and ten of the complete set of different categories of antibiotics trialed). On the other hand, nineteen strains (42.2%) showed resistance to four antibiotics, thirteen strains (28.9%) to five, and a further thirteen strains had a multidrug-resistant (MDR) phenotype, there being resistance to six antibiotics in six strains, to seven in four, to eight in two, and to nine in one, as may be seen from Figure 9.

### 3.7. Relationships between Virulence Factors

To determine any possible relationship between the three groups of factors tested that might influence the virulence of *L. monocytogenes*, the presence of virulence genes, the potential for biofilm formation, and resistance to antibiotics, a Pearson correlation analysis was performed. The results obtained show a weak but non-significant (*p* > 0.05) correlation between the percentage of resistant reactions per isolate and the potential for biofilm formation (OD_580_ values) (r = 0.227;), and between the potential for biofilm formation and the percentage of virulence genes detected (r = 0.152). No correlation was observed between the percentage of strains with resistance to antibiotics and the percentage of virulence genes detected (r = 0.098).

## 4. Discussion

### 4.1. Prevalence of Listeria spp. and Listeria monocytogenes

The average percentage of samples contaminated with *Listeria* spp. was 70.0%; this figure being as high as 100% in three of the nine establishments studied. This widespread presence may be related to the ubiquity of these bacteria and to fecal contamination during evisceration, since birds can be carriers of this microorganism [37,38]. The prevalence of *Listeria* spp. found in this research work was higher than that observed in other studies, where values noted were 14.6% in poultry meat products [39], 22.0% in meat and poultry carcasses [40], 40.0% in chicken carcasses [41] and 48.0% in fresh chicken meat [42]. However, the results presented here are very similar to those observed in other studies carried out in north-western Spain: 73.0% in poultry preparations [12], 76.3% in free-range poultry meat [43], 92.1% in raw chicken meat [37] and 95.0% in chicken carcasses [26].

The predominant species in the present study was *L. monocytogenes*, present in 50% of the samples analyzed. This percentage is lower than those previously recorded in the North-West of the Iberian Peninsula for prepared poultry meat, with figures of 56.0% [12] and 70% [44] having been reported. The higher prevalence obtained in meat preparations may be due to the greater amount of handling that such samples receive, as suggested previously by Rodríguez-Melcón et al. [44]. On the other hand, the prevalence detected during the research work being presented here was higher than values previously observed in fresh chicken meat from this region, with figures of 24.5% [37] and of 32% [26] having been seen, and in work undertaken by other authors elsewhere, most ranging from 0% to 20% [39,42,45,46,47,48,49,50,51,52,53].

It should be noted that various outlets (E2, E4 and E5) provided samples completely uncontaminated with *L. monocytogenes*, while in others (E3 and E8) the microorganism was detected in more than 85.0% of the items acquired. This fact may be related to the ability of *L. monocytogenes* to form biofilms and so resist the usual treatments employed to eliminate it, giving it the capacity to remain in food-processing environments for long periods, causing cross-contamination [54]. However, these results should be interpreted with caution due to the low number of samples analyzed.

After *L. monocytogenes*, *L. innocua* was the most prevalent species, being detected in 36.7% of the samples. This value is similar to the 46.3% found in poultry meat by Fallah et al. [47] and the 32.0% in poultry meat preparations recorded by Panera-Martínez et al. [12]. It should be noted that in some previous studies, *L. innocua* was the most prevalent species, with percentages for contamination of 57.8% [26] and 59.5% [37] in samples of fresh chicken and of 67.4% in poultry meat samples [43]. In contrast, other authors have detected a lower prevalence of *L. innocua* in poultry meat products, with 0% being observed by Osaili et al. [50] and 13.6% by Amajoud et al. [39].

The remaining *Listeria* species studied had low prevalence. For instance, *L. grayi* was found in 6.7% of the samples, a value slightly higher than the 2.0% observed in previous studies carried out on poultry meat preparations by Panera-Martínez et al. [12], the 2.2% seen in fresh chicken meat by Alonso-Hernando et al. [37] and the 3.5% in processed chicken meat products recorded by Osaili et al. [50]. No strains of *L. seeligeri* or *L. ivanovii* were isolated, this being a result similar to those of other authors, who found a low prevalence of these species, ranging between 0.0% and 1.8% [39,43,50] for *L. seeligeri*, and between 0.0% and 1.1% for *L. ivanovii* [37,43].

Finally, three isolates obtained from one of the samples analyzed proved impossible to identify at the species level. A similar outcome was noted in earlier work with regard to strains obtained from whole carcasses and legs of poultry, with figures of 6.7% in 1993 and 12.8% in 2006 [37].

### 4.2. Concentration of Viable Cells of Listeria monocytogenes

The levels of *L. monocytogenes* in the samples were determined by q-PCR. The use of this technique yielded positives in 20 samples (66.7%). The levels of total cells, both viable and inactivated, ranged between 2.40 and 5.22 log_10_ cfu/g, while for viable cells alone, they ran from <2.15 to 3.93 log_10_ cfu/g. The concentrations of *L. monocytogenes* were similar to those obtained in previous studies carried out on poultry meat preparations, at between 2.34 and 5.96 log_10_ cfu/g for total cells [12] and on minced chicken meat, running between <2.15 and 3.25 log_10_ cfu/g for viable cells, and between <2.15 and 4.32 log_10_ cfu/g for total cells [44].

Treatment with PMA brought about a decrease in the levels of *L. monocytogenes* detected. This was a finding to be expected, since the compound in question binds to the DNA of damaged cells only, preventing amplification, which thereafter solely affects the DNA from intact cells [55]. In eleven of the twenty samples with *L. monocytogenes* detected by q-PCR, the fluorescence detection limit was not breached after PMA treatment. In these cases, the number of viable cells was deemed to be below the limit of detection (2.15 log_10_ cfu/g). This detection limit in itself was lower than that set by other authors, for instance, the 3 to 4 log_10_ cfu/g used by Rantsiou et al. [56].

There has been much research focusing on the detection of *Listeria* spp. and of *L. monocytogenes* in food, using both culture-dependent (plating) and culture-independent (q-PCR) methods. However, the investigation being described here differs from most of those studies, which were based on previous enrichment stages, and which ruled out any possibility of quantifying the pathogen. The concentrations of *L. monocytogenes* noted in the present study were higher than those observed by other authors in raw chicken meat, with levels detected by plate count not exceeding a figure of 3 log_10_ cfu/g [53] or, when they were higher, this affected only a very small percentage of samples [57]. The differences between those research works and the present study may be due to the fact that the plate counts that were performed by the researchers in question were able to quantify only viable culturable cells, whereas the v-PCR technique permits non-culturable viable cells to be counted as well [58,59].

### 4.3. Comparison between OCLA&PCR and v-PCR

The high score for sensitivity obtained with v-PCR (100%) as against OCLA&PCR, seen as the “gold standard”, points to an absence of false negatives in this culture-independent method. In other words, all the samples rated positive using the OCLA&PCR technique were also classed as positive by v-PCR. In contrast, the figure for specificity, 66.7%, was an outcome of the presence of five samples classed negative by plating (OCLA&PCR) but positive by v-PCR. If it is kept in mind that the v-PCR procedure applied gives very good results in pure cultures of *L. monocytogenes*, and in samples inoculated with known amounts of the pathogen [15,58], it is highly likely that these five samples contained viable but non-culturable *L. monocytogenes* cells, so that no growth was observed on the culture medium, but they were detected by v-PCR.

### 4.4. Virulence Genes

All the *L. monocytogenes* isolates had between six and nine of the virulence genes *hlyA*, *actA*, *inlA*, *inlB*, *inlC*, *inlJ*, *prfA*, *plcA* and *iap*. These genes are frequently found in strains of *L. monocytogenes* of food origin. Thus, Arslan and Baytur [27] detected all nine of the virulence factors listed in 100% of the *L. monocytogenes* strains they isolated from fresh beef and poultry meat. For their part, Anwar et al. [60] also observed high prevalences for these genes: *hlyA* (100%), *actA* (100%), *inlA* (100%), *inlB* (100%), *inlC* (90.2%) and *inlJ* (90.2%), *prfA* (100%) and *plcA* (100%).

The presence of multiple virulence factors in *L. monocytogenes* strains is related to their potential to cause disease, since they favor entry into eukaryotic cells [60]. Of the virulence factors studied, *inlA*, *inlB*, *inlC* and *inlJ* are genes that encode proteins of the internalin family and are associated with penetration by the microorganism into non-phagocytic cells [61,62,63]. Moreover, it is noteworthy that all the isolates had the *actA* gene, which encodes a surface protein associated with the passing of the bacterium from one cell into another without contact with the extracellular medium, allowing *L. monocytogenes* to evade a host’s immune system [64,65].

The *hlyA* (alpha-hemolysin) and *iap* (invasion-associated protein) genes were found in 100% of the isolates analyzed. In contrast, the *plcA* (phosphatidylinositol-specific phospholipase C) and *prfA* (positive regulatory factor A) genes appeared in only 55.6% of the isolates. These four genes are predictors of the major virulence factors in *L. monocytogenes*, since they are directly related to its pathogenicity [66,67] and to the control of the expression of other virulence genes in this bacterium [68].

### 4.5. Potential for Biofilm Formation

The potential of *L. monocytogenes* isolates to form biofilm was limited. Isolates were classified as non-biofilm producers (33.3%), or as weak (51.1%), moderate (2.2%) or strong (13.3%) biofilm producers. Other authors have obtained results similar to those from the present study. Thus, Kayode and Okoh [69] isolated from ready-to-eat products a number of *L. monocytogenes* strains, 69.1% of which had some potential for biofilm formation. For their part, several researchers [70,71,72] have observed a clear predominance of strains with a weak or moderate capacity to form biofilms in *L. monocytogenes* isolates from food or clinical samples, these results being consistent with those of the work presented here. On the contrary, other authors have detected a strong ability to form biofilm in *L. monocytogenes* [69,73,74,75]. On these lines, various studies carried out in recent years revealed that 100% of the isolates of *L. monocytogenes* investigated were capable of forming biofilms on the surfaces tested [6,72,76,77,78]. Among other factors, such discrepancies between studies may be due to the varying origins of isolates, the employment of different strains, or the use of differing techniques, surface materials, or both.

One striking fact is that all the strains classified as strong or moderate biofilm producers (15.5%) were isolated from wings or drumsticks from establishments E3, E6 or E8. Indeed, the isolates from establishment E8 had a high average value of OD_580_ (1.73 ± 0.84). The greater capacity to form biofilm found among the strains isolated in samples from outlet E8 may be an outcome of cross-contamination produced by contact with the surfaces in that establishment, since biofilms are a frequent cause of contamination of food during processing [6].

### 4.6. Antibiotic Resistance

The susceptibility of the 45 *L. monocytogenes* isolates from cuts of poultry was tested against 15 antibiotics of clinical interest. All the isolates presented either resistance or reduced susceptibility to OX, FOX, CTX, FEP and CIP. Percentages clearly over 50% were observed for F (55.3%) and ENR (51.1%). For the remaining antibiotics, the percentages of strains that were resistant or had reduced susceptibility varied between 0.0% and 22.2%. In previous studies carried out with poultry meat preparations [12] and with poultry carcasses and portions [37,79], strains of *L. monocytogenes* resistant to these antibiotics were also found. This fact is worrying, since some of these substances are used for the treatment of listeriosis.

The extent of prevalence of susceptibility to ampicillin, to which 100% of strains were susceptible, is reassuring, since this is the drug of first choice, whether used alone or in combination with gentamycin, in treating human listeriosis [79]. These data are fully consistent with previous studies carried out on strains isolated from chicken meat from north-western Spain, in which no strain with reduced susceptibility or susceptibility to ampicillin was found [37]. In addition, among alternatives for the treatment of listeriosis would be the administration of erythromycin, vancomycin, trimethoprim-sulfamethoxazole, rifampicin, tetracycline and chloramphenicol [79]. These are drugs that in the present study met with some resistance or reduced susceptibility in isolates, with figures ranging between 2.38% and 52.38% of the total.

There was a high average number of resistances per strain: 5.11 ± 1.30 when resistance in the strict sense was considered, and 6.91 ±1.59 when resistance and reduced susceptibility were taken together. This shows a significant increase in comparison with previous studies, in which the mean values for resistance to antibiotics were 1.60 in 1993 and 4.24 in 2006 [37]. Hence, the results of the research being reported here confirm an increase in prevalence in *L. monocytogenes* of resistance to antibiotics occurring over recent years [47]. This would appear to be due primarily to the acquisition of mobile genetic elements, such as plasmids or transposons, through horizontal transfer mechanisms [80].

The high prevalence of antibiotic-resistant bacteria in foodstuffs of animal origin, which was observed in most of the research works performed in the last years, is most likely related to the use of antibiotics in animal production. This has had a great impact on microbial populations and has triggered the selection and proliferation of resistant bacteria, as has previously been suggested [81]. It must be noted that a great prevalence of resistance to antibiotics widely employed in animal production has been observed in the present work [82,83,84]. The resistance to substances whose use has been prohibited for decades in food-producing animals (e.g., nitrofurantoin) might be due to mechanisms for cross-resistance or co-resistance, which may have contributed to the persistence over time of genes for resistance to this compound [85,86].

The considerable levels of resistance observed in this study are a cause for concern, since resistance to antibiotics compromises the usefulness of these compounds as a therapeutic option, representing a significant challenge for public health [87]. On these lines, it should be noted that infections by multi-resistant bacteria not only increase morbidity and mortality rates, but also cause a considerable increase in the costs arising from medical treatments [88,89].

### 4.7. Relationships between Virulence Factors

The correlation values observed between the variables studied were low, with only weak values being found for the links between the percentage of resistance to antibiotics of each isolate and its potential for biofilm formation (OD_580_) (r = 0.227), and between the potential for biofilm formation and the percentage of virulence genes detected (r = 0.152). So, it cannot be stated that the greater the potential for biofilm formation, the greater also the percentage of resistance to antibiotics. These results do not coincide with what was expected, since the formation of biofilms promotes resistance to antibiotics in bacteria. In addition, biofilms are an environment that favors the transfer of genes and the appearance of adaptive mutations [90,91,92].

On the other hand, no correlation was demonstrated between the percentage of resistance to antibiotics and that for virulence genes in the isolates (r = 0.098). It should be noted that higher correlation values were expected, since the three variables in question are directly related to the pathogenicity of *L. monocytogenes* [23,93].

## 5. Conclusions

Raw poultry meat is an important reservoir of *L. monocytogenes*, in terms both of prevalence and of concentration. The v-PCR method is a useful technique for the rapid detection and quantification of *L. monocytogenes* in food. Additionally, it allows a distinction to be made between total and viable cells, whether culturable or not, thus making it possible to determine the concentration of bacteria with the potential to cause illness. On the other hand, the presence in chicken meat of strains with a high prevalence of virulence genes, with potential for biofilm formation, and with multiple resistances to antibiotics are worrying in the context of food safety and public health. The results from this study emphasize how crucial it is to maintain correct hygiene practices during the processing of poultry, so as to prevent retail establishments from becoming a reservoir of *L. monocytogenes* strains with a strong potential for virulence. Furthermore, it is vital to avoid undercooking chicken meat and to prevent cross-contamination events in order to reduce the risk of human listeriosis.

## Figures and Tables

**Table 1:** List of primers used, showing sequence, annealing temperature and size of amplified fragment [9].

Gene	Primer	Sequence (5′ → 3′)	Temp. (°C)	Product Size (pb)
*lmo1030*	F	GCTTGTATTCACTTGGATTTGTCTGG	62	509
	R	ACCATCCGCATATCTCAGCCAACT		
*lin0464*	F	CGCATTTATCGCCAAAACTC	60	749
	R	TCGTGACATAGACGCGATTG		
*oxidoreductase*	F	GCGGATAAAGGTGTTCGGGTCAA	62	201
	R	ATTTGCTATCGTCCGAGGCTAGG		
*lmo0333*	F	GTACCTGCTGGGAGTACATA	58	673
	R	CTGTCTCCATATCCGTACAG		
*namA*	F	CGAATTCCTTATTCACTTGAGC	58	463
	R	GGTGCTGCGAACTTAACTCA		
*prs*	F	GCTGAAGAGATTGCGAAAGAAG	58	370
	R	CAAAGAAACCTTGGATTTGCGG		

**Table 2:** List of primers used in detecting virulence factors, their sequence, the size of the amplified fragment, and concentrations used [27].

Gene	Primer	Sequence (5′ → 3′)	Product Size (bp)	Concentration (µM)
*hlyA*	F	CCTAAGACGCCAATCGAA	702	0.50 each
	R	AAGCGCTTGCAACTGCTC		
*actA*	F	GCTGATTTAAGAGATAGAGGAACA	827	0.50 each
	R	TTTATGTGGTTATTTGCTGTC		
*inlB*	F	CTGGAAAGTTTGTATTTGGGAAA	343	0.50 each
	R	TTTCATAATCGCCATCATCACT		
*inlA*	F	ACGAGTAACGGGACAAATGC	800	0.25 each
	R	CCCGACAGTGGTGCTAGATT		
*inlC*	F	AATTCCCACAGGACACAACC	517	0.20 each
	R	CGGGAATGCAATTTTTCACTA		
*inlJ*	F	TGTAACCCCCGCTTACACAGTT	238	0.15 each
	R	AGCGGCTTGGCAGTCTAATA		
*plcA*	F	CTGCTTGAGCGTTCATGTCTCATCCCCC	1484	0.20 each
	R	CATGGGTTTCACTCTCCTTCTAC		
*prfA*	F	CTGTTGGAGCTCTTCTTGGTGAAGCAATCG	1060	0.20 each
	R	AGCAACCTCGGTACCATATACTAACTC		
*iap*	F	ACAAGCTGCACCTGTTGCAG	131	0.50 each
	R	TGACAGCGTGTGTAGTAGCA		

**Table 3:** Thermocycling programs used in amplification reactions.

Programs	Denaturation	Cycles				Elongation
*hlyA*	94 °C/5 min	30×	94 °C/30 s	50 °C/45 s	72 °C/90 s	72 °C/5 min
*actA*	94 °C/2 min	40×	94 °C/180 s	53 °C/60 s	72 °C/120 s	72 °C/5 min
*inlB*	94 °C/2 min	35×	94 °C/45 s	60 °C/45 s	72 °C/90 s	72 °C/8 min
*inlA*, *inlC* e *inlJ*	94 °C/2 min	30×	94 °C/20 s	55 °C/20 s	72 °C/50 s	72 °C/2 min
*plcA* y *prfA*	95 °C/2 min	35×	95 °C/15 s	60 °C/30 s	72 °C/90 s	72 °C/10 min
*iap*	95 °C/2 min	35×	95 °C/15 s	60 °C/30 s	72 °C/90 s	72 °C/10 min

**Table 4:** Results of quantification by q-PCR for total cells and viable cells from samples with *Listeria monocytogenes*. The samples are ordered from the lowest to highest concentration of viable cells.

	Total Cells (without PMA)			Viable Cells (with PMA)			%Viable Cells	OCLA&PCR
Sample	Ct	ng DNA	Log_10_ cfu/g	Ct	ng DNA	Log_10_ cfu/g		
Breast1	39.24	0.000006	2.40	>40	<0.000003	<2.15	56.4	+
Wing1	38.98	0.000007	2.49	>40	<0.000003	<2.15	46.3	+
Breast2	38.70	0.000008	2.58	>40	<0.000003	<2.15	37.5	+
Thigh1	38.65	0.000009	2.60	>40	<0.000003	<2.15	36.1	+
Wing2	38.62	0.000009	2.61	>40	<0.000003	<2.15	35.3	+
Breast3	38.60	0.000009	2.61	>40	<0.000003	<2.15	34.8	−
Thigh2	38.04	0.000014	2.80	>40	<0.000003	<2.15	22.8	−
Wing3	37.89	0.000015	2.85	>40	<0.000003	<2.15	20.4	−
Wing4	37.67	0.000018	2.92	>40	<0.000003	<2.15	17.3	+
Breast4	36.14	0.000058	3.42	>40	<0.000003	<2.15	5.4	+
Drumstick1	36.13	0.000058	3.42	>40	<0.000003	<2.15	5.4	+
Wing5	37.44	0.000022	2.99	39.99	0.000003	2.16	14.6	−
Drumstick2	37.87	0.000016	2.85	39.95	0.000003	2.17	20.8	+
Wing6	37.67	0.000018	2.92	39.66	0.000004	2.27	22.3	+
Wing7	38.61	0.000009	2.61	38.89	0.000007	2.52	81.0	−
Drumstick3	33.12	0.000567	4.41	38.09	0.000013	2.78	2.3	+
Drumstick4	34.63	0.000181	3.91	37.44	0.000022	2.99	12.0	+
Drumstick5	34.50	0.000199	3.96	36.36	0.000049	3.35	24.6	+
Drumstick6	35.23	0.000115	3.72	36.11	0.000059	3.43	51.7	+
Drumstick7	30.66	0.003628	5.22	34.57	0.000190	3.93	5.2	+

**Table 5:** Antibiotic resistance patterns in forty-five *Listeria monocytogenes* isolates from cuts of poultry.

Pattern (Number of Isolates)	Antibiotic														
	AMP	OX	FOX	CTX	FEP	CN	E	VA	SXT	RD	TE	C	CIP	ENR	F
1 (19)	S	**R**	**R**	**R**	**R**	S	S	S	S	S/I *	S	S	I	S/I *	S/I *
2 (1)	S	**R**	**R**	**R**	**R**	S	S	S	**R**	I	S	S	I	I	I
3 (6)	S	**R**	**R**	**R**	**R**	S	S	S	S	**R**	S	S	I	S/I *	S/I *
4 (3)	S	**R**	**R**	**R**	**R**	S	S	S	S	S/I *	S	S	**R**	S/I *	S/I *
5 (1)	S	**R**	**R**	**R**	**R**	S	S	S	S	S	S	S	I	**R**	S
6 (2)	S	**R**	**R**	**R**	**R**	S	S	S	S	S	S	S	I	S/I *	**R**
7 (1)	S	**R**	**R**	**R**	**R**	S	**R**	S	S	I	S	S	I	I	**R**
8 (1)	S	**R**	**R**	**R**	**R**	S	S	S	S	**R**	S	S	**R**	I	I
9 (1)	S	**R**	**R**	**R**	**R**	S	S	S	**R**	**R**	S	S	I	I	I
10 (1)	S	**R**	**R**	**R**	**R**	S	S	S	S	**R**	S	S	I	S	**R**
11 (2)	S	**R**	**R**	**R**	**R**	S	S	S	**R**	S/I *	S	S	I	I	**R**
12 (1)	S	**R**	**R**	**R**	**R**	S	S	S	**R**	I	S	S	**R**	I	**R**
13 (1)	S	**R**	**R**	**R**	**R**	S	**R**	S	**R**	**R**	I	S	I	I	S
14 (2)	S	**R**	**R**	**R**	**R**	S	S	S	**R**	**R**	S	S	**R**	I	S
15 (1)	S	**R**	**R**	**R**	**R**	S	**R**	S	S	**R**	S	S	**R**	I	**R**
16 (1)	S	**R**	**R**	**R**	**R**	S	**R**	S	**R**	**R**	S	S	I	I	**R**
17 (1)	S	**R**	**R**	**R**	**R**	R	S	S	**R**	**R**	S	S	**R**	I	**R**

AMP (ampicillin, 10 µg), OX (oxacillin, 1 µg), FOX (cefoxitin, 30 µg), CTX (cefotaxime, 30 µg), FEP (cefepime, 30 µg), CN (gentamycin, 10 µg), E (erythromycin, 15 µg), VA (vancomycin, 30 µg), SXT (trimethoprim-sulfamethoxazole, 25 µg), RD (rifampicin, 5 µg), TE (tetracycline, 30 µg), C (chloramphenicol, 30 µg), CIP (ciprofloxacin, 5 µg), ENR (enrofloxacin, 5 µg), F (nitrofurantoin, 300 µg). R = resistant; I = intermediate (reduced susceptibility); S = susceptible. *, different results were observed for the different isolates in each pattern.

## Data Availability

The data presented in this study are available on request from the corresponding author. The data are not publicly available due to confidentiality issues.
